# Subsiding of Periodontitis in the Permanent Dentition in Individuals with Papillon-Lefèvre Syndrome through Specific Periodontal Treatment: A Systematic Review

**DOI:** 10.3390/healthcare10122505

**Published:** 2022-12-10

**Authors:** Dagmar Schnabl, Felix Maximilian Thumm, Ines Kapferer-Seebacher, Peter Eickholz

**Affiliations:** 1 Department of Prosthetic Dentistry, Medical University Innsbruck, 6020 Innsbruck, Austria; 2 Department of Conservative Dentistry and Periodontology, Medical University Innsbruck, Anichstraße 35, 6020 Innsbruck, Austria; 3 Department of Periodontology, Center for Dentistry and Oral Medicine (Carolinum), Johann Wolfgang Goethe-University Frankfurt/Main, 60596 Frankfurt, Germany

**Keywords:** Papillon–Lefèvre syndrome, Haim–Munk Syndrome, periodontitis, antibiotics, periodontal treatment

## Abstract

Papillon–Lefèvre syndrome (PLS) is a rare hereditary disease characterized by palmoplantar hyperkeratosis (PPK) and periodontitis in the primary and permanent dentition, usually resulting in edentulism in youth. Subsiding of PLS-associated periodontitis through specific therapy has occasionally been reported. We aimed to systematically assess periodontal treatment strategies that may decelerate disease progression. A systematic literature search was conducted at PubMed/LIVIVO/Ovid (Prospero registration number CRD42021223253). Clinical studies describing periodontal treatment success—defined as loss of ≤four permanent teeth because of periodontitis and the arrest of periodontitis or probing depths ≤ 5 mm—in individuals with PLS followed up for ≥24 months. Out of the 444 primarily identified studies, 12 studies reporting nine individuals were included. The timely extraction of affected or, alternatively, all primary teeth, compliance with oral hygiene instructions, supra- and subgingival debridement within frequent supportive periodontal care intervals, and—in eight patients—adjunctive systemic antibiotic therapy (mostly amoxicillin/metronidazole) effected a halt in disease progression. The suppression of *Aggregatibacter actinomycetemcomitans* below the detection limit was correlated with the subsiding of periodontitis. Successful controlling of PLS-associated periodontitis may be achieved if high effort and patient compliance are provided.

## 1. Introduction

Papillon–Lefèvre syndrome (PLS) is a rare autosomal recessive disease with a prevalence of 1 in 4,000,000 at birth [1]. Haim–Munk syndrome (synonyms: Cochin Jewish disorder and Congenital Keratosis) is an extremely rare variant of PLS that has only been seen in descendants of an inbred Jewish family originally from Cochin, India, who migrated to Israel [1]. Cardinal features are palmoplantar hyperkeratosis (PPK) and severe periodontal destruction of the primary and permanent dentition. The first dermatological and periodontal manifestations commonly emerge in toddlerhood [2]. After a proper eruption of the primary teeth, gingivitis occurs around the age of two to three years, and subsequent periodontitis leads to alveolar bone resorption, attachment loss, and premature loss of the primary dentition [2,3]. In permanent dentition, the destructive process recurs, usually leading to complete tooth loss in youth.

Periodontitis is a chronic inflammatory disease that is triggered by dysbiotic bacterial deposits on teeth (dental biofilm) and it destroys the tooth supporting tissues (connective tissue attachment and bone) [4,5]. The interface of the tooth surface and oral mucosa (i.e., gingiva) is a weak spot of the body’s integrity. Dental biofilm accumulating at the gingival margin may enter between the tooth surface and gingiva into the connective tissue or even bone. As a respective defense mechanism, the body gathers inflammatory cells in the affected gingiva to prevent the microbiota from entering. Some asaccharolytic and iron-dependent bacteria (e.g., Porphyromonas gingivalis) may, on the one hand, trigger inflammation to thrive on proteins of the inflammatory gingival fluid, but, on the other hand, evade the respective immune reaction (dysbiosis) maintaining chronic inflammation [5]. Certain periodontal bacteria, such as *Aggregatibacter actinomycetemcomitans* (*A. actinomycetemcomitans*), are strongly associated with destructive inflammatory responses [6]. Inflammatory and immunological defense may be disturbed by genetic factors (e.g., PLS) that deteriorate the function of cells and mediators. In most people, highly prevalent periodontitis progresses slowly and starts to manifest at a middle age [7]. In rare monogenic disorders such as PLS, genetic factors severely affect immunity, and rapidly progressing periodontitis is likely to appear early in life. Thus, the treatment of periodontitis aims to prevent biofilm accumulation on tooth surfaces (effective individual oral hygiene) and to remove the biofilm from periodontal pockets (subgingival instrumentation) [8].

Homozygous or compound heterozygous loss-of-function mutations of the *cathepsin C* gene, encoding dipeptidyl-aminopeptidase *cathepsin* C (*CTSC*), are associated with PLS [1,9,10]. *CTSC*, a lysosomal cysteine protease, impacts epidermal differentiation and scaling and is expressed in epithelial regions typically affected in PLS patients. Furthermore, the cysteine protease—through the removal of N-terminal inhibitory peptides—activates certain serine proteases (e.g., *proteinase 3* (*PR3*)) in immunologic cells, such as neutrophil granulocytes, cytotoxic lymphocytes, natural killer cells, alveolar macrophages, and mast cells, which participate in cell-mediated phagocytosis, cytotoxicity, and activation of various inflammatory mediators [1,9,10]. *PR3* activates *cathelicidin LL-37* (*LL-37*), known as a potent antimicrobial to most strains of *A. actinomycetemcomitans*. Consequently, loss-of-function mutation of *CTSC* in PLS resulting in a lack of *PR3* leads to the deficit of antimicrobial and immunomodulatory functions of *LL-37* in the gingiva, allowing for infection with *A. actinomycetemcomitans* and contributing to the development of severe periodontal disease [11]. *A. actinomycetemcomitans* cannot reliably be suppressed below levels of detection using a subgingival instrumentation alone [12,13,14]. Thus, the adjunctive use of systemic antibiotics is recommended. The combination of amoxicillin and metronidazole has been shown to be effective at suppressing *A. actinomycetemcomitans* below the limits of detection [15]. A loss of *CTSC* function increases susceptibility to infections frequently seen in PLS patients, e.g., furunculosis, pyodermia, liver abscess, pneumonia, and others [3,10,16,17]. In some PLS patients, intracranial calcifications and mental retardation were reported [3]. The likely pathomechanism of periodontal destruction with PLS is a reduced immune response due to inadequate chemotaxis, phagocytosis, and bactericidal activity of the granulocytes, a diminished number of T-helper and regulatory cells, and a decreased proliferation of lymphocytes [18,19,20,21]. While disease progression seems default in most affected individuals, occasional cases with loss-of function mutations in *CTSC* displaying PPK, but no periodontitis, have been reported. Furthermore, PLS cases with successful periodontal treatment have been documented. To the best of the authors’ knowledge, up until now, interventional studies on periodontal treatment of PLS-associated periodontitis do not exist.

The aim of this systematic review was a compilation of reported PLS cases with successfully arrested periodontitis to identify effective periodontal treatment strategies.

## 2. Materials and Methods

### 2.1. Protocol and Registration

The systematic search was performed according to the Preferred Reporting Items for Systematic Review and Meta-Analysis (PRISMA) guidelines [22] and was registered at Prospero under CRD42021223253.

A structured approach was used to formulate the research question for this systematic review using five components, commonly known by the acronym “PICOS”: the patient population (P), the interventions (I), the comparison group (C), the outcome of interest (O), and the study design (S). The respective PICOS components for this review are:

P: Subjects with PLS who have completed at least one cycle of non-surgical periodontal therapy rendering clinical success;

I: Non-surgical/surgical periodontal treatment;

C: PLS patients without periodontal treatment;

O: Loss of less than four permanent teeth because of periodontitis and arrest of clinical attachment loss with probing depths ≤ 5 mm at the end of a determined follow-up interval of ≥24 months;

S: Clinical trials, case-control studies, cross-sectional studies, cohort studies, case series, and case reports published in peer-reviewed scientific journals.

### 2.2. Literature Search Strategy

The systematic search was accomplished in the electronic databases PubMed, LIVIVO, and Ovid through thr use of the following keywords: (Papillon–Lefèvre OR “papillon lefevre” OR Haim–Munk OR “Haim Munk” OR palmoplantar kerato*) AND (Cathepsin C OR CTSC). The publication date was open to the past. The determined cut-off date was 31 March 2021. Additionally, a manual search was conducted, including browsing of the reference lists of the selected studies.

### 2.3. Screening and Selection

Clinical studies of successful periodontal treatment of adult individuals clinically and/or genetically diagnosed with PLS were included. Children were included if the follow-up interval extended after the eruption of all permanent incisors and first molars. Treatment success was evaluated at a follow-up-interval of ≥ 24 months, and was defined as loss of less than four permanent teeth because of periodontitis, corresponding to a remainder of at least 24 permanent teeth in the absence of dental aplasia (third molars excluded), ensuring a proper chewing function and posterior tooth support. According to the S3 clinical practice guideline of the European Federation of Periodontology, endpoint of periodontal therapy was defined when probing depths ≤ 5 mm were achieved [8]. (Moderately deep pockets are considered manageable by subgingival instrumentation during supportive periodontal care.) Studies in English or German, available in full text, were included.

The following types of studies were considered: clinical trials, case-control studies, cross-sectional studies, cohort studies, case series, and case reports published in peer-reviewed scientific journals. Excluded studies were cell culture laboratory studies, animal studies, narrative reviews, and questionnaire studies. Titles and abstracts were checked regarding the pre-defined eligibility criteria. Abstracts with unclear methodology were included in full-text assessment to avoid the exclusion of potentially relevant articles.

#### 2.3.1. Assessment of Heterogeneity

The heterogeneity of the included studies was evaluated based on following factors: (1) study design and (2) subjects’ characteristics.

#### 2.3.2. Quality Assessment

The Joanna Briggs Institute (Adelaide, Australia) quality assessment tool [23] was applied. Each study was classified into the following groups: low risk of bias if all quality criteria were judged as “present”, moderate risk of bias if one or more key domains were “unclear”, and high risk of bias if one or more key domains were “absent”. Only low risk of bias studies were eligible.

### 2.4. Data Extraction

The extracted main outcomes in the studies reporting successful periodontal treatment in PLS were as follows: (1) clinical and genetic diagnosis of PLS; (2) age at baseline; (3) initial dental, periodontal parameters, and microbiological assessment, if available; (4) description of disease progression and applied therapies; and (5) outcome and follow-up.

## 3. Results

### 3.1. Study Selection

**Figure 1:**
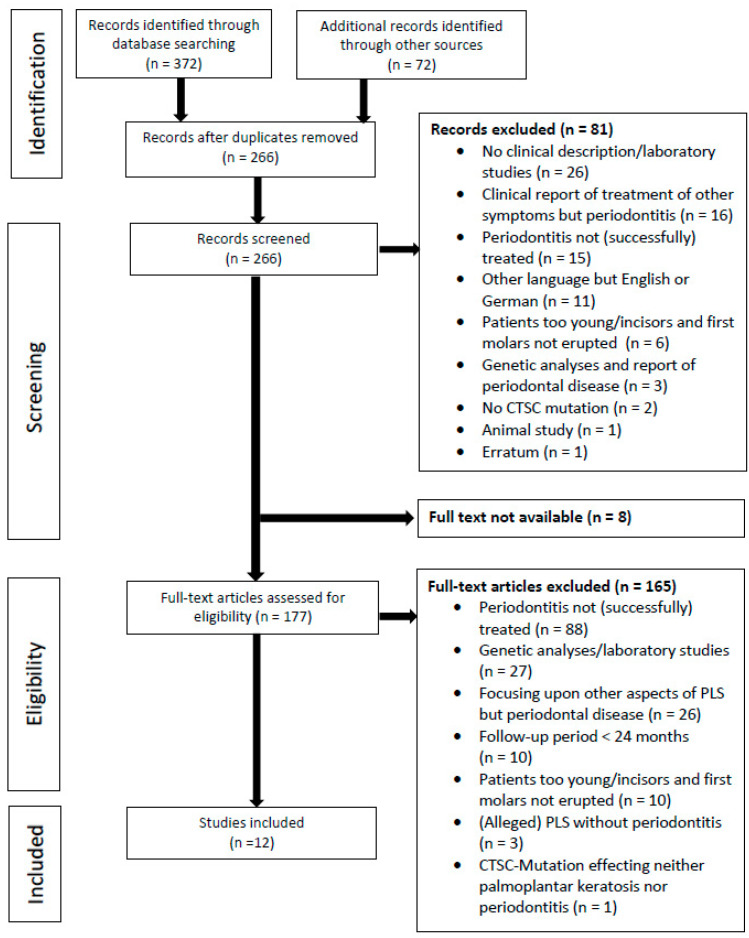
PRISMA flow diagram illustrating the literature search and selection process.

The literature search flowchart is presented in Figure 1. Out of 266 identified studies (after removal of the duplicates), 81 were excluded based on the title and abstract. Of these, 26 studies were laboratory studies, 16 reported the treatment of other symptoms but periodontitis, 15 reported periodontitis that was not (successfully) treated, 11 were written in another language besides English or German, 6 described the periodontal treatment of children with a follow-up interval ending before the eruption of incisors/first molars, 3 presented genetic analyses in individuals diagnosed with periodontitis and severe tooth loss, 2 described other pathologies than PLS, 1 was an animal study, and 1 was an erratum.

Of the remaining 185 studies, eight were not available in full text, seven of which published between 1965 and 1997 and one abstract of an oral presentation delivered in the year 2005. One hundred and seventy-seven articles were screened by full text. Eighty eight studies reported individuals diagnosed with periodontitis who received no or unsuccessful periodontal treatment, twenty seven were genetic analyses/laboratory studies, 26 twenty six with other aspects of PLS than the periodontal condition, ten documented a follow-up period of <24 months after periodontal treatment, ten reported on young children with permanent incisors and first molars not yet erupted, three studies reported PLS cases (two alleged and one genetically confirmed) without signs of periodontitis [24,25,26], and one reported a *CTSC* mutation not effecting PLS (neither PPK nor PD) [27].

Finally, 12 studies were included [2,28,29,30,31,32,33,34,35,36,37,38]. Eight of these were case series [2,28,29,30,33,34,35,38] and four were case reports [31,32,36,37].

### 3.2. Subjects, Diagnosis, and Therapy

Twelve of the included studies reported on a total of nine individuals (six males and three females) meeting the inclusion criteria. Multiple reports existed of four individuals (identification numbers (IDs) 4, 5, 6, and 7). In these cases, previous reports of early treatment were included in synopsis, with subsequent reports of treatment success after an overall follow-up of ≥24 months.

Table 1 presents an overview of the subjects’ characteristics, periodontal treatment measures, and follow-up period of nine included individuals [2,28,29,30,31,32,33,34,35,36,37,38]. In four individuals, PLS was confirmed by genetic examination (ID 6: homozygous variants in c.755 A>T, p.Q252L; ID 7: compound heterozygous variants in c.947 T>G, p.L316R and c.1268 G>C, p.W423S [29]; ID 8: homozygous variants in c.854 C>T; p.P285L [29]; ID 9: compound heterozygous variants in c.322A>T, p.Lys108Ter and c.504C>G, p.Tyr168Ter). In the remaining subjects, PLS diagnosis was based on clinical symptoms and positive family history.

The following treatment steps were unanimously considered essential in the reduction of the probing depths and periodontal inflammation by authors reporting successful periodontal therapy: (I) strict biofilm management including repeated oral hygiene instructions and chlorhexidine mouth rinses; (II) professional supra- and subgingival debridement within a stringent supportive periodontal therapy with intervals ranging from one week up to three months; and the (III) extraction of severely affected teeth. Only the dentist of patient ID 9 chose to not extract severely affected primary teeth [38]. In this patient, primary teeth exfoliated at the usual age and, intriguingly, the permanent dentition was not affected by periodontitis by the age of 18 years, despite poor biofilm management at the time.

In seven patients, initial microbiological testing was performed and was positive for *A. actinomycetemcomitans* and/or other periopathogenic bacteria (Table 2). In seven patients, antibiotic courses were conducted, and in six the suppression of *A. actinomycetemcomitans* below the level of detection after treatment was documented and related with the arrest of periodontitis [28,29,33,34,35,36,37]. In three individuals, all of the primary teeth were extracted, inducing an edentulous period of nine months to two years until the eruption of the permanent teeth [2,28,31,32]. The authors emphasized the benefit of a complete (temporary) extinction of microbiological niches with regard to the elimination of periopathogens that did not (re-) occur in the permanent dentition. In one individual (ID 6), treatment success was accomplished by the extraction of only severely affected primary teeth, together with mechanical cleaning and antibiotic therapy [33,34].

## 4. Discussion

Numerous case reports have recounted the default progression of periodontal destruction and the failure of periodontal treatment in individuals afflicted with PLS. The aim of this systematic review was to identify promising therapeutic strategies by detecting and analyzing case reports considering the successful treatment of PLS-associated periodontitis.

Regarding treatment success, we deliberately set tight inclusion criteria (requiring an arrest of periodontitis implying steadily reduced probing depths of maximally 5 mm for 24 months and a maximum loss of four permanent teeth, third molars excluded, due to periodontitis) with respect to favorable esthetics and proper masticatory function. In all of the included reports, conformingly, periodontal treatment comprised classical mechanical debridement (when necessary, under general anesthesia), repeated oral hygiene instructions, chlorhexidine mouth rinses, the extraction of severely affected primary and/or permanent teeth, and a strict and frequent recall interval. In the majority of studies, microbiological testing for *A. actinomycetemcomitans* and/or other periopathogenic bacteria was performed before and after treatment. The suppression of *A. actinomycetemcomitans* below the level of detection through mechanical treatment with adjunctive systemic antibiotic courses was correlated with the subsiding of periodontitis [28,29,33,34,35,36,37].

For antibiotic therapy, a combination of amoxicillin and metronidazole, as used in individuals ID 4, 6, 7, 8, and 9, has been shown to be a commendable strategy [29,33,34,35,36,37,38]. In individual ID 3, after unsuccessful courses of minocycline and erythromycin, according to antibiogram, ofloxacin proved to be effective in the suppression of *A. actinomycetemcomitans* below the level of detection [28]. Individual ID 1, after an edentulous interval through the extraction of all primary teeth, was administered repeated courses of tetracycline to prevent the onset of periodontitis in permanent teeth [2]. The administration of antibiotics (mostly initially and at recurrence of periodontitis or *A. actinomycetemcomitans*) varied from one week to two years [2,28,29,31,32,33,34,35,36,37,38].

In three cases, the complete extraction of the primary dentition effecting an edentulous period of several months (with or without the delivery of intermediate full dentures) was suspected to have eliminated all periopathogenic bacteria and facilitated the eruption of permanent teeth in a steadily physiological microbiological environment [2,28,31,32]. In individual ID 6 (as well as his younger brother, who was excluded from this review because of the incomplete eruption of incisors at the end of the study period), the extraction of only severely affected primary teeth, together with mechanical cleaning and antibiotic therapy, effected treatment success [33,34]. Whenever permanent teeth were already erupted in the presence of severe periodontitis of the primary teeth, they were usually also afflicted with periodontitis and in need of periodontal therapy or, eventually, extraction [28,35,36,37].

Intensive maintenance therapy and compliance with homecare seem to constitute key success factors [35]. Lux et al. (2005), in the description of the course of disease of individual ID 6, demonstrated a deterioration of periodontal parameters after 60 months of successfully controlled periodontitis when, the patient’s compliance with home care decreased, which emphasized the necessity for a continuous motivation and recall of PLS patients [36]. In individual ID 6, fixed orthodontic therapy was required and included the extraction of two central mandibular incisors with severe alveolar bone loss. Originally, all four mandibular incisors were scheduled for extraction. However, the patient’s mother insisted on the retention of these teeth. The result of the orthodontic treatment was satisfactory, but the long-term prognosis of the remaining mandibular incisors was rated as critical due to severe clinical attachment loss of up to 6 mm, and isolated probing depths of 4 mm with bleeding on probing remaining after 79 months of continuous monitoring and treatment [36]. Seventeen years after active treatment, both incisors still were present [35]. Nickles et al. (2013) also reported orthodontic treatment in individual ID 7 [35], and Wiebe et al. (2001) considered orthodontics following the successful periodontal therapy of individual ID 4 [31]. This is in accordance with the recently published periodontal practice guidelines, which state that orthodontic therapy has no detrimental effects on periodontal conditions in periodontitis patients with a healthy but reduced periodontium, provided the results of the periodontal therapy are maintained during the active orthodontic treatment phase [39].

More than 75 different mutations in the *CTSC* gene may cause PLS, Haim–Munk syndrome, or prepubertal periodontitis, which are subsumed to the same phenotypical spectrum [40,41]. PLS manifestation seems to range in the severity and progression of tissue destruction and age of disease onset. Atypical phenotypes of *CTSC* mutations with palmoplantar hyperkeratosis but lack of periodontal involvement and vice versa have been described [24,25,26,38]. Atypical phenotypes also may occur within one molecular family. Soskolne et al. (1996) reported on an individual without periodontal involvement who had two (out of five) siblings that suffered from both PPK and periodontitis with complete tooth loss in teenage years, and a second molecular family with three siblings exhibiting PPK plus periodontitis, but one brother displaying periodontitis only [24]. The basis of phenotypic variation is genetic variability between individuals in conjunction with acquired risk factors. The authors suspect the influence of certain genetic or epigenetic mechanisms to cause a variable or discordant expression of cardinal features of PLS, such as the co-expression of linked loci or non-linked modifying genes, compound heterozygosity, or genomic imprinting (gene silencing) in homozygous cases with absent consanguinity, or also occasional manifestations of heterozygosity [24,26]. Genetic variability probably also influences the periodontal treatment success of PLS.

The identified treatment strategies and the proposed treatment goal of the suppression of *A. actinomycetemcomitans* must be investigated in larger cohorts of PLS patients. The European research project “Solving the unsolved Rare Diseases (RD)”, which was funded under the European Union’s Horizon 2020 research and innovation program, aims to solve large numbers of RDs and to improve the diagnostics and therapy of RD patients. The resolution of the remaining open questions with PLS will primarily rely on the standardization of the diagnostic criteria. Clinical centers that specialize in this rare pathology need to apply congruent approaches for the exact characterization of clinical features in conjunction with genetic validation, which should be reached without exception in all patients and relevant family members [42].

In conclusion, only a few case reports on the successful treatment of PLS associated periodontitis exist. The early diagnosis of PLS and the suppression of *A. actinomycetemcomitans* below the detection level might be critical factors for treatment success. Suppression of *A. actinomycetemcomitans* may be achieved through extraction of (affected or all) primary teeth, preferably before the eruption of permanent teeth and/or supra- and subgingival debridement with adjunctive systemic antibiotic treatment with amoxicillin and metronidazole. The suppression of *A. actinomycetemcomitans* in conjunction with frequent supportive periodontal care, preferably carried out by a specialized dentist or team, seems to enable a delay or halt of the usual default disease progression. Successful controlling of PLS-associated periodontitis implies a high effort and compliance within a stringent treatment concept.

## Figures and Tables

**Table 1:** Subjects’ characteristics including gender, age at the end of follow-up, periodontal therapy, and follow-up interval of included cases with successful periodontal treatment. Clinical diagnosis of Papillon–Lefèvre syndrome was based on severe periodontitis in early childhood and palmoplantar hyperkeratosis in all of the included individuals. Successful periodontal treatment was defined as the loss of less than four permanent teeth because of periodontitis and arrest of periodontitis with steady probing depths ≤ 5 mm for ≥24 months. Multiple reports existed from individuals with IDs 4, 5, 6, and 7.

Reference	ID	Age (y)	Gender	Periodontal Therapy	Adjunctive Antibiotic Treatment	Additional Therapeutic Interventions	Follow-Up (y)
Preus et al. (1987) [2]	1	9	male	supra- and subgingival debridement, OHI, 0.2% chlorhexidine mouthrinse extraction of all primary teeth	tetracycline 250 mg/d for 2–4 weeks intermittently in case of suspected gingival abnormality and 0.25 mg/day continuously for 2 y	none	5
Ishikawa et al. (1994) [28]	2	9	female	extraction of all primary teeth	none	full dentures	5
Ishikawa et al. (1994) [28]	3	12	female	supra- and subgingival debridement, OHI, flap surgery	minocycline 200 mg/d for 2 weeks erythromycin 1 g/d for 1 month ofloxacin 300 mg/day for 1 month	none	5
Rüdiger et al. (1999) [29] Kressin et al. (1995) [30]	4	11	female	supra- and subgingival debridement, OHI Meridol^®^ mouth rinse extraction of two mandibular incisors 0.06% chlorhexidine jet irrigation	amoxicillin 750 mg/d plus clavulanic acid and metronidazole 375 mg/d for 1 week	removable partial denture	6
Wiebe et al. (2001) [31] French et al. (1995) [32]	5	17	male	supra- and subgingival debridement, OHI 0.12% chlorhexidine mouthrinse extraction of all primary teeth	2 rounds of metronidazole	full dentures	12
Nickles et al. (2011) [33] Schacher et al. (2006) [34]	6	10	male	supra- and subgingival debridement, OHI extraction of severely affected primary teeth 1% chlorhexidine gel 0.12% chlorhexidine mouthrinse	amoxicillin 750 mg/d and metronidazole 750 mg/d for 1 week	none	5
Nickles et al. (2013) [35] Lux et al. (2005) [36] Eickholz et al. (2001) [37]	7	24	male	supra- and subgingival debridement, OHI 0.1% chlorhexidine mouthrinse e 1% chlorhexidine gel 30% chlorhexidine chips in 4 teeth	amoxicillin 750 mg/d and metronidazole 500 mg/d for 1 week	orthodontics	17
Nickles et al. (2013) [35]	8	17	male	supra- and subgingival debridement, OHI extraction of severely affected teeth	amoxicillin 750 mg/d plus clavulanic acid and metronidazole 600 mg/d for 1 week repeated in case of recurrence	orthodontics	13
Umlauft et al. (2021) [38]	9	18	male	supra- and subgingival debridement, OHI 0.2% chlorhexidine mouthrinse	amoxicillin and metronidazole	none	15

ID, identification number; y, years; OHI, repeated oral hygiene instructions; et al., et al. teri; d, day(s).

**Table 2:** Microbiolocigal findings in patients with Papillon–Lefèvre syndrome. The presence of *Aggregatibacter actinomycetemcomitans*, *Porphyromonas gingivalis, Prevotella intermedia, Fusobacterium nucleatum, Campylobacter rectus*, and *Eikenella corrodens* before and/or after periodontal therapy was evaluated.

Reference	ID	Microbiological Testing Baseline	Antibiotics	Extraction of All Primary Teeth	Presence of *A. actinomycetemcomitans* after Therapy
Preus et al. (1987) [2]	1	not evaluated	Tetracycline	yes	not evaluated
Ishikawa et al. (1994) [28]	2	*A. actinomycetemcomitans* *P. gingivalis * *P. intermedia * *F. nucleatum * *C. rectus * *E. corrodens*	None	yes	negative after extraction of all primary teeth
Ishikawa et al. (1994) [28]	3	*A. actinomycetemcomitans* *P. gingivalis * *P. intermedia * *F. nucleatum * *C. rectus * *E. corrodens*	minocycline erythromycin ofloxacin	no	positive after minocycline positive after erythromycin negative after ofloxacine
Rüdiger et al. (1999) [29] Kressin et al. (1995) [30]	4	*A. actinomycetemcomitans* *P. gingivalis*	amoxicillin and metronidazole	no	negative after 3 y
Wiebe et al. (2001) [31], French et al. (1995) [32]	5	*P. gingivalis * *T. forsythia*	None	yes	not evaluated
Nickles et al. (2011) [33] Schacher et al. (2006) [34]	6	*A. actinomycetemcomitans* *P. gingivalis * *T. forsythia * *T. denticola*	amoxicillin and metronidazole	no	negative 1 and 5 y
Nickles et al. (2013) [35] Lux et al. (2005) [36] Eickholz et al. (2001) [37]	7	*A. actinomycetemcomitans* *P. gingivalis * *P. intermedia * *F. nucleatum * *C. rectus * *E. corrodens*	amoxicillin and metronidazole	no	negative after 7 months, 2, 3, and 4 y positive after 7 y negative after 8 and 12 y
Nickles et al. (2013) [35]	8	*A. actinomycetemcomitans*	amoxicillin/clavulanic acid and metronidazole initially and at recurrences after 3 and 8 y	no	positive after 3 and 8 y negative after 9 y
Umlauft et al. (2021) [38]	9	not evaluated	amoxicillin and metronidazole	no	not evaluated

ID, identification number; et al., et al.; y, years.

## Data Availability

All data analyzed for this SR is publicly available in the respective articles that are referred to.
